# Solubility of Methane in Water: Some Useful Results for Hydrate Nucleation

**DOI:** 10.1021/acs.jpcb.2c04867

**Published:** 2022-10-12

**Authors:** Joanna Grabowska, Samuel Blazquez, Eduardo Sanz, Iván M. Zerón, Jesús Algaba, José Manuel Míguez, Felipe J. Blas, Carlos Vega

**Affiliations:** † Departamento Química Física I, Fac. Ciencias Químicas, Universidad Complutense de Madrid, 28040 Madrid, Spain; ‡ Department of Physical Chemistry, Faculty of Chemistry and BioTechMed Center, Gdansk University of Technology, ul. Narutowicza 11/12, 80-233 Gdansk, Poland; § Laboratorio de Simulación Molecular y Química Computacional, CIQSO-Centro de Investigación en Química Sostenible and Departamento de Ciencias Integradas, Universidad de Huelva, 21006 Huelva, Spain

## Abstract

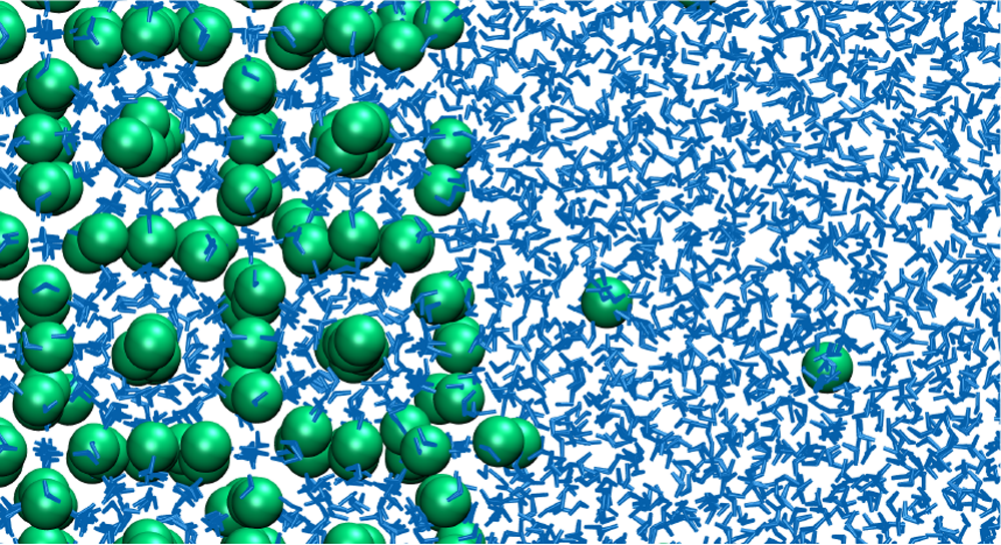

In this paper, the solubility of methane in water along the 400 bar isobar is determined by computer simulations using the TIP4P/Ice force field for water and a simple LJ model for methane. In particular, the solubility of methane in water when in contact with the gas phase and the solubility of methane in water when in contact with the hydrate has been determined. The solubility of methane in a gas–liquid system decreases as temperature increases. The solubility of methane in a hydrate–liquid system increases with temperature. The two curves intersect at a certain temperature that determines the triple point *T*_3_ at a certain pressure. We also determined *T*_3_ by the three-phase direct coexistence method. The results of both methods agree, and we suggest 295(2) K as the value of *T*_3_ for this system. We also analyzed the impact of curvature on the solubility of methane in water. We found that the presence of curvature increases the solubility in both the gas–liquid and hydrate–liquid systems. The change in chemical potential for the formation of hydrate is evaluated along the isobar using two different thermodynamic routes, obtaining good agreement between them. It is shown that the driving force for hydrate nucleation under experimental conditions is higher than that for the formation of pure ice when compared at the same supercooling. We also show that supersaturation (i.e., concentrations above those of the planar interface) increases the driving force for nucleation dramatically. The effect of bubbles can be equivalent to that of an additional supercooling of about 20 K. Having highly supersaturated homogeneous solutions makes possible the spontaneous formation of the hydrate at temperatures as high as 285 K (i.e., 10K below *T*_3_). The crucial role of the concentration of methane for hydrate formation is clearly revealed. Nucleation of the hydrate can be either impossible or easy and fast depending on the concentration of methane which seems to play the leading role in the understanding of the kinetics of hydrate formation.

## I Introduction

Hydrates are compounds formed when water is in contact with a gas phase of a small molecule (i.e., methane or carbon dioxide) at moderate to high pressures and at low temperatures.^1^ The molecules of water form an open structure, and the guest molecules of the gas occupy the lattice positions (although in nature, the gas hydrates are not completely occupied). The simplest hydrate structure is denoted as sI^1^ and is the one formed by methane and carbon dioxide. The unit cell of the sI solid belongs to the cubic system. Hydrates are of interest both from a fundamental point of view and from a practical point of view.^1^ Hydrates are found close to the shores of the coast, and one could obtain natural gas from them. From a fundamental point of view, they are formed from a mixture of rather small and simple molecules.

Over the past few years, we have studied in detail the nucleation in a number of systems.^2−6^ We have implemented the technique of seeding^4^ which allows one to estimate nucleation rates by combining simulation results and classical nucleation theory. The technique of seeding has been applied successfully to a number of systems from simple ones such as hard spheres (HS) and Lennard-Jones (LJ) to more complex such as water or salty water,^7^ obtaining good agreement with results obtained from more rigorous techniques. It would be interesting to extend the methodology of seeding to more complex systems, as is the case of hydrates. In fact, in 2012, Molinero et al. implemented the technique of seeding^8^ to obtain a first estimate of the nucleation rate of hydrates using the mW model of water.^9^

Before considering nucleation using seeding, there are some issues that should be solved. First of all, the estimate of the temperature at a certain pressure at which the three phases can coexist is needed. Conde and Vega^10^ suggested in 2011 to use direct coexistence of the three phases to estimate *T*_3_. It was observed that combining TIP4P/Ice^11^ and a simple LJ model for methane gives the estimates of *T*_3_ that are in good agreement with the experimental ones (the same was found by Miguez et al. in a later study on carbon dioxide hydrates^12^). Notice that, to obtain values of *T*_3_ in good agreement with experiment, water models with a good prediction of the experimental melting point of ice Ih are required.^13^ After this study, a number of groups have revisited the problem using the same force field.^14−18^ Although the agreement between different groups is reasonable, some differences still exist, with values of *T*_3_ at 400 bar being in the range of 290–300 K for this force field. It would be of interest before addressing nucleation studies to reach some consensus on the value of *T*_3_ for this system.

The solubility of methane in water increases as the temperature decreases. This has been studied both in experiments and in simulation.^19,20^ However, the solubility of methane from the hydrate is rarely studied either in experiments or in simulations.^21^ In this work, we shall perform a study of the solubility of the hydrate. It will be shown that solubility studies allow us to estimate *T*_3_. Thus, in principle, the values of *T*_3_ obtained from the direct coexistence of the three phases and those of the solubility should be coincident. We shall show that this is the case and that they are the same to within the estimated uncertainty which is of about 2 K.

A key quantity in classical nucleation theory (CNT) is the driving force for nucleation, which is denoted as *Δμ*_nucleation_. This is the change in chemical potential of the “reaction” where a molecule of the hydrate is formed from the molecules of methane and water both in the aqueous solution. Although it would be possible to determine *Δμ*_nucleation_ from experiments, often this is not possible due to the lack of information about the thermodynamic properties of the system (i.e., enthalpies, volumes, etc.). Estimates of *Δμ*_nucleation_ are rare with the exception of the work of Kashchiev and Firoozabadi.^22,23^ In this work, we shall address the issue of the evaluation of *Δμ*_nucleation_ using two slightly different thermodynamic routes. Results of both routes will be coincident (within the combined error bar).

An interesting issue is whether one can modify the driving force for nucleation artificially.^24^ The analysis reveals that the concentration of methane dramatically affects the value of *Δμ*_nucleation_. In general, increasing the concentration of methane increases the driving force for nucleation. We found, in agreement with the work of other authors,^25,26^ that bubbles increase the solubility of methane, and we will quantitatively estimate the impact of this increment in solubility on the driving force for nucleation. It will be shown that bubbles increase the driving force, but there is a limit, as it is not possible to have bubbles which are mechanically stable below a certain size of about 1.2 nm radius. However, we will show that another strategy is possible by generating homogeneous supersaturated solutions in which the nucleation of methane hydrates can occur even at temperatures just 10 K below *T*_3_. Also, it will be shown that the solubility of methane in water when the solution is in contact with the hydrate increases by introducing curvature in the interface.

Thus, in this paper, we address the first steps toward addressing the issue of the homogeneous nucleation of hydrates under experimental conditions with special interest in *T*_3_ and in *Δμ*_nucleation_. It can be said that the study of the solubility of methane in water (either from the gas or from the hydrate) contains a lot of interesting physics and the key ingredients to understand hydrate nucleation in future studies following the increasing activity in the field of the last years.^8,27−41^

## II Methods

All of the results presented in this work were obtained using classical molecular dynamics (MD). Simulations were performed using the GROMACS package^42,43^ in the *NpT* ensemble. Three types of barostats were used depending on the problem. Either isotropic *NpT* (where the three sides of the simulation box change proportionally), anisotropic *NpT* (where each side changed independently), or *Np*_*z*_*T* (where only one size of the simulation box was allowed to fluctuate) simulations will be performed in this work. A time step of 2 fs was used. To keep the temperature constant, the Nosé–Hoover thermostat^44,45^ was employed, with a coupling constant of 2 ps. The pressure was kept constant with the use of the Parrinello–Rahman barostat,^46^ and the time constant used was equal to 2 ps. For all of the simulations, the pressure was equal to 400 bar. For electrostatic and van der Waals interaction, a cutoff of 9 Å was used. Coulombic interactions were treated using the PME method.^47^ Long-range energy corrections to energy and pressure were included for the Lennard-Jones part of the potential. For water, the TIP4P-Ice^11^ water model was used, while, for methane, the parameters were taken from refs (48) and (49). For cross-interaction between TIP4P-Ice water and methane models, Lorentz–Berthelot rules were applied. In order to maintain the geometry of water molecules in the systems, the LINCS algorithm^50,51^ was employed.

For simplicity throughout this paper, the phase of pure methane will be denoted as “gas phase”, as in the literature one often uses the term “gas hydrates” even though methane is studied under supercritical conditions. In certain cases, we have simulated the solid phase of the hydrate with structure sI (*Pm*3̅*n*) with a lattice constant of about 12 Å. In this solid,^52^ one has 8 molecules of methane and 46 molecules of water in the unit cell (i.e., the ratio of water to methane molecules is 5.75). The 8 molecules of methane occupy the 8 cavities available in the solid structure (2 of them being smaller than the other 6). Oxygens occupy the crystallographic positions *c*(6), *k*(24), and *i*(16), whereas the methanes occupy the *d*(6) and *a*(2) crystallographic positions. We used full occupancy (i.e., all cages are occupied by methane molecules). Experimentally occupancies around 95% are often found.^1,53^ In the hydrate structure, one finds proton disorder. Proton disordered configurations satisfying the Bernal–Fowler rules^54^ were generated using the algorithm of Buch et al.^55^

To analyze the melting of the hydrate or its stability with time, we shall determine the size of the largest solid cluster of the hydrate (i.e., we shall compute the number of molecules of water forming the hydrate). For this purpose, we shall use the order parameter proposed by Lechner and Dellago.^56^ We found that  was a reasonable order parameter (notice that for ice Ih we used^57^, but this is not a good order parameter for hydrate formation, as noted by Algaba et al.^58^). While  may not be the optimal order parameter, as many other choices have been proposed for hydrates,^59−61^ it is reasonable, simple, and adequate for our purpose of detecting molecules of the solid phase. Details about the implementation of  and the threshold value used to label a molecule as solid are provided in the Supporting Information.

Since the solubility of methane in water is small, we shall use the unsymmetrical convention^62^ in the thermodynamic description of mixtures so that we will have a dominant component (solvent) which in this case will be water and a minor component (solute) which in this case will be methane. The chemical potential of water in the mixture is given by^63^
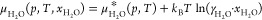
1where *x*_H_2_O_ is the molar fraction of water, μ_H_2_O_^*^(*T*, *p*) (i.e., the standard state for water) is the chemical potential of pure water at the same *T* and *p* of the mixture and *k*_B_ is the Boltzmann constant . γ_H_2_O_ is the activity coefficient of water. The chemical potential of methane in the aqueous solution is given by
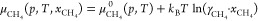
2where μ_CH_4__^0^(*T*, *p*) is the standard state of methane which depends on *T* and *p* but not on composition. Notice that the standard state of water is a “real” state, as it corresponds to pure water at the same *T* and *p*. However, the standard state of methane is a “virtual” state, as it does not correspond to any physical realization. It corresponds to a “virtual” state of pure methane where intermolecular interactions are identical to those obtained at infinite dilution. From a statistical mechanics point of view, μ_CH_4__^0^(*p*, *T*) is related to the residual chemical potential of methane in water at infinite dilution (after adding the constant *k*_B_*T* ln(ρ_H_2_O_·Λ_CH_4__^3^·*q*_CH_4__), where ρ_H_2_O_ is the number density of pure water, Λ_CH_4__ is the thermal de Broglie wavelength of methane, and *q*_CH_4__ is the ideal gas partition function of methane containing all degrees of freedom but translation) and can also be related to the Henry constant.^64^ In the unsymmetrical convention, it holds that when *x*_H_2_O_ → 1 both γ_H_2_O_ and γ_CH_4__ go to 1.^62^ Since the solubility of methane in water is quite small, it is reasonable (although not exact) to assume that the activity coefficients for both methane and water are close to 1. We shall assume that this is the case in our “approximate” treatment of the aqueous solution of methane.

## III Results and Discussion

### III.A Solubility of Methane in Water from the Gas Phase

By using the direct coexistence method, we shall compute the solubility of methane in water for several temperatures along the 400 bar isobar. For that purpose, a slab of water (2959 molecules) was put in contact with a slab of gas (methane, 922 molecules) with an initial size of the simulation box of around 3.6 × 3.6 × 14.4 nm^3^. The planar interface is located in the *XY* plane, and pressure is applied perpendicular to the interface using the *Np*_*z*_*T* ensemble. Simulations were run for more than 500 ns, and averages were obtained using the last 250 ns (typically 100–150 ns were required to reach the equilibrium concentration). From the simulations, we also obtained the surface tension of the water–methane interface using the pressure tensor:^65^
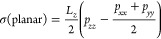
3Results for the surface tension are presented in Table 1. Notice that our results are only qualitative, since, to obtain precise values of this magnitude, a large cutoff and/or the inclusion of long-range corrections are needed. Using the methodology of Lundberg and Edholm^66^ (which has been shown to work properly by Blazquez et al.^67^), we roughly estimated a long-range correction of about 2 mJ/m^2^. Moreover, for some temperatures, we repeated the runs using a much larger cutoff (i.e., 17 Å) and some results are also presented in Table 1. It is confirmed that the values of σ for a larger cutoff are around 1.5–2 mJ/m^2^ higher than those presented in Table 1 for the cutoff used in this work (i.e., 9 Å).

**Table 1:** Interfacial Free Energy σ (for a Planar Interface) between Methane Gas and an Aqueous Solution at *p* = 400 bar Obtained at Different Temperatures^a^

*T* (K)	σ (mJ/m^2^)	σ* (mJ/m^2^)
250	62.8	
260	61.8	63.1
270	61.5	
280	61.0	62.2
290	60.4	
300	59.6	61.4
310	59.1	

a Values of σ are in mJ/m^2^. The values with an asterisk were obtained using a cutoff of 17 Å. The error of the values of σ is of about 0.4 mJ/m^2^.

**Figure 1:**
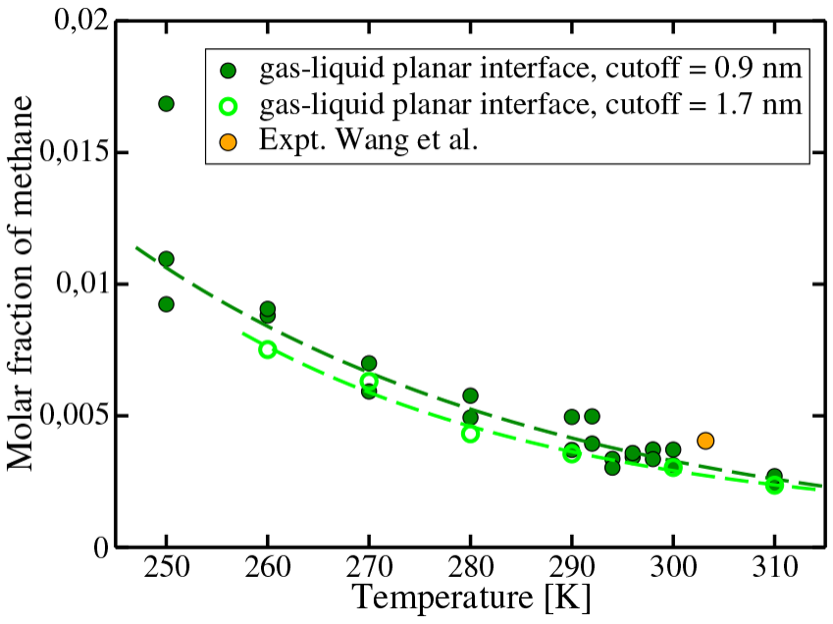
Solubility of methane in water when the solution is in contact with the gas phase at *p* = 400 bar. For each *T*, the results of two (three in the case of 250 K) independent runs are shown to provide some idea of the uncertainty of our calculations. For six temperatures (open green circles), we computed the solubility using a larger cutoff (i.e., 17 Å). For comparison, an experimental value of the solubility of methane in water at 303 K of Wang et al.^68^ is also presented (orange point). Dashed curves are a guide to the eye.

In Figure 1, the solubilities of methane in water along the isobar are shown. As can be seen, the solubility decreases as the temperature increases. The solubility is small, and the molar fraction of methane was never larger than 0.02. We did not observe the formation of hydrate in any of the runs. Additionally, we evaluated the solubility at six temperatures using a larger cutoff (i.e., 17 Å). As can be seen, the use of a larger cutoff reduces slightly (by 5–10%) the solubility of methane in the aqueous phase. It should be mentioned that the solubility of water in the methane gas is negligible. To a good approximation, the gas phase is pure methane, and in this work, we have not computed the solubility of water in methane, since it is at least 1 order of magnitude lower than that of methane in water.

In Figure 1, the experimental value of the solubility of methane in water at a temperature of 303 K and a pressure of 400 bar is also shown.^68^ As can be seen, the value that we obtained reproduces the experimental value reasonably well. It is true, however, that the agreement between simulation and experimental results could be improved by introducing a small scaling factor to the default Lorentz–Berthelot combination rule.

It goes without saying that, when the system reaches equilibrium, the temperature in the two phases is the same, the pressure is the same (as one has a planar interface), and the chemical potential of methane in water is the same, so that it holds that
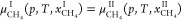
4
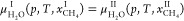
5where the superscripts I and II label the two phases at equilibrium. Since we are dealing with a two-component system having methane and water, the molar fraction of one of them (for instance, methane) is enough to describe the composition of the mixture (as the sum of the molar fraction of methane and water is 1). There is an interesting consequence of eqs 4 and 5. As the gas phase is basically pure methane and as the chemical potential is the same in the two phases, one can obtain easily the chemical potential of methane in the water solution by computing that of pure methane in the gas phase (i.e., neglecting the very small presence of water in the gas phase). Therefore, we shall use the approximation
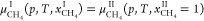
6where phase I is the aqueous phase and phase II is the gas phase (which we shall assume is pure methane).

The change in the chemical potential of methane along an isobar can be obtained from the thermodynamic relation:
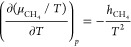
7

**Figure 2:**
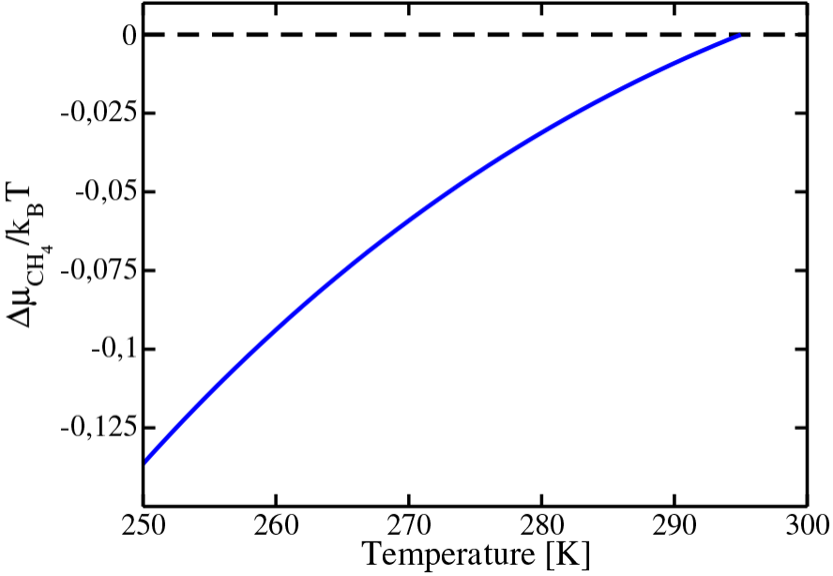
Chemical potential of bulk methane obtained along the isobar *p* = 400 bar. We arbitrarily set the value of the chemical potential to zero at the temperature *T* = 295 K.

For reasons that will be clear later, let us set the chemical potential of pure methane at 400 bar to zero at a certain reference temperature *T*_ref_. Then, we can integrate the previous equation to obtain
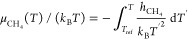
8where μ_CH_4__ is the chemical potential of methane and *h*_CH_4__ is its partial molar enthalpy (either per molecule when using *k*_B_ in the denominator or per mole when using the ideal gas constant *R* in the denominator). Of course in the case of a pure substance, the partial molar enthalpy is simply the molar enthalpy. By performing simulations of pure methane along the 400 bar isobar, we can compute the enthalpy, and using the previous equation, we can compute the chemical potential of methane. We shall not include the kinetic energy when computing the enthalpy (i.e., 3/2 *k*_B_*T* in the case of methane), as this term cancels out when computing chemical potential differences evaluated at constant *T* and *p*. Results for the chemical potential of methane as a function of *T* are presented in Figure 2 (where we have selected for reasons that will be discussed later *T*_ref_ = 295 K).

An interesting question is whether there is any possible temperature limit to perform the simulations to compute the solubility. As we did not observe nucleation of the hydrate, the simulations can be performed at any temperature without any difficulty, although of course as the temperature decreases the dynamics slows down and equilibration is more difficult.

### III.B Solubility of Methane in Water from the Hydrate Phase

We shall also compute the solubility of methane in water when the solution is in contact with the hydrate along the 400 bar isobar. For that purpose, we shall use the direct coexistence method. A slab of the hydrate will be put in contact with water solution. The dimensions of the simulation box were 4.8 × 4.8 × 8.7 nm^3^ (with about 2500 molecules of water in the hydrate and 3500 molecules of water in the solution and with a total number of 445 molecules of methane, most of them (but fifteen) in the hydrate). We used complete occupancy of the methane in the cages of the hydrate structure. Again, the interface was located in the *XY* plane and the *Z* axis was perpendicular to the interface. An anisotropic barostat was applied along the three axes so that the dimensions of the simulation box could change independently. The pressures used were identical and equal to 400 bar in all three directions. The anisotropic barostat was important to remove any stress in the solid and to obtain the correct solubility. Simulations were performed for temperatures in the range 250–330 K. The length of the simulations was greater than 500 ns in all cases. The averages were obtained after removing the first 250 ns. It should be mentioned that to evaluate the solubility of methane in water when in contact with the hydrate it is convenient to use an initial configuration with a concentration of methane in water not too far from the equilibrium value to reach equilibrium as fast as possible. For all temperatures, we performed two runs, the first one to estimate the value of the solubility and the second one to determine it with high accuracy. When equilibrium is reached, the chemical potential of the two components (water and methane) is the same in the two phases, the temperature is the same, and the pressure is the same (as we have a planar interface).

**Figure 3:**
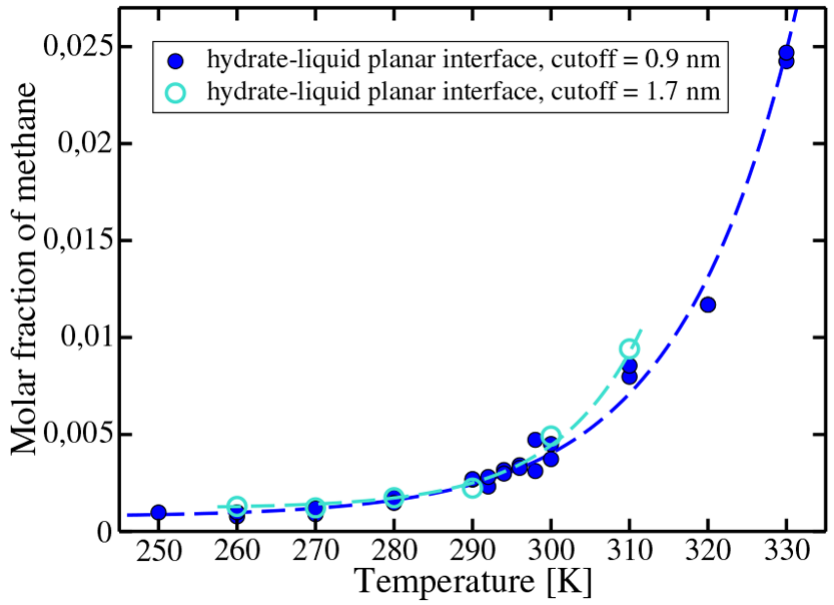
Solubility of methane in water when in contact with the hydrate phase at *p* = 400 bar. For six temperatures (open blue circles), we computed the solubility using a larger cutoff (i.e., 17 Å). Dashed curves are a guide to the eye.

Results for the solubility of methane when solution is in contact with the hydrate are presented in Figure 3. As can be seen, the solubility of methane increases with temperature. We were able to obtain the solubility from low temperatures up to a temperature of 330 K. At a temperature of 340 K, it was not possible to determine the solubility, since the hydrate melted. The melting was a two-step process. First, in the liquid phase, a bubble of pure methane nucleated spontaneously. After that, the methane of the aqueous solution moved quickly to the bubble, and the methane from the hydrate moved to the aqueous solution, provoking the melting of the hydrate. Thus, there is a kinetic limit at high temperatures to determine the solubility of methane from the hydrate.

Some plots of the solubility of the hydrate as a function of temperature (for pressures different from the one considered in this work) were described in an experimental paper^21^ but to the best of our knowledge were never reported in simulations.

### III.C Three-Phase Coexistence from Solubility Calculations

**Figure 4:**
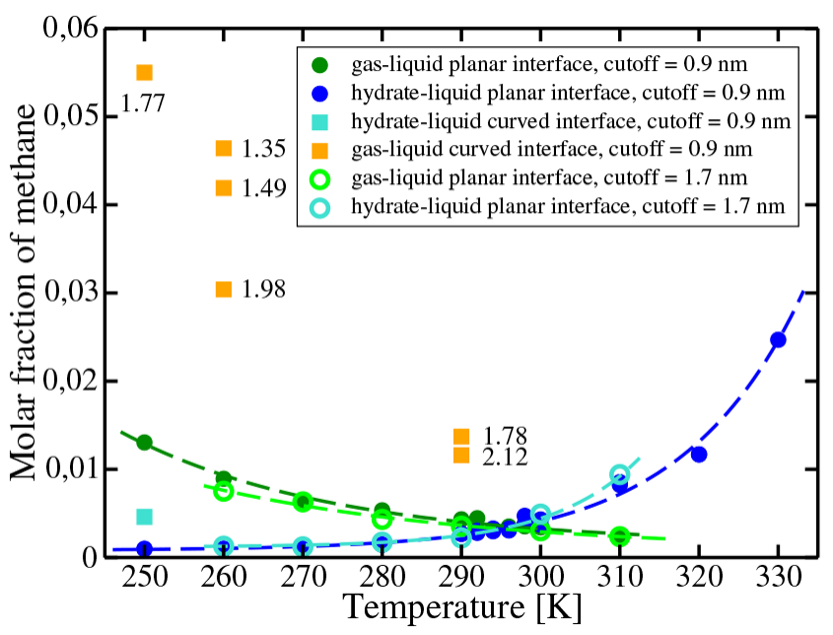
Solubilities of methane from the gas and from the hydrate along the isobar *p* = 400 bar. The crossing of the two curves determines the triple point temperature *T*_3_ at 400 bar. The solubilities of methane when in contact with bubbles of methane of different radius (given in nm) are also shown as orange squares. For six temperatures, we computed the solubilities of methane from the gas phase (open green circles) and hydrate (open light blue circles) using a larger cutoff (i.e., 17 Å). The light blue square is the solubility of methane when the hydrate is forming a spherical cluster. Dashed curves are a guide to the eye.

It is now interesting to plot both solubility curves (the solubility of methane in water from the gas and the solubility of methane in water from the hydrate) in the same plot. This is done in Figure 4. As can be seen, there is a temperature at which these solubility curves cross. At the crossing point, the aqueous phase has the same composition and it is in equilibrium simultaneously with the gas phase and with the hydrate. Therefore, it is a triple point where the three phases are at equilibrium. This temperature will be denoted as *T*_3_. To the best of our knowledge, this is the first time *T*_3_ is determined from solubility calculations of two independent two-phase coexistence runs. However, this method is routinely used in the context of the classical equations of state to determine three-phase coexistence conditions for gas and/or solid phases.^69−71^

The value obtained from our calculations for *T*_3_ is 297(2) K. Our value is consistent with the values reported by other groups using the three-phase coexistence approach.^10,14,16,18^ A comprehensive study of the impact of the cutoff of the potential on *T*_3_ is beyond the scope of this work, as it will require using a much larger cutoff or even to use PME methods to properly account for the LJ interactions and that will make the calculations terribly expensive. However, we have included in Figure 4 the solubilities of methane from the gas phase in water and from the hydrate phase in water at several temperatures using a much larger cutoff (i.e., 17 Å). The solubility of methane from the hydrate is hardly affected by the value of the cutoff, whereas that from the gas phase is reduced, indicating that the impact of the truncation of the potential is more important when the two phases differ significantly in density. With the larger cutoff, the intersection of the two solubility curves occurs at 295(2) K.

The summary is that solubility calculations are also a (relatively simple) route to *T*_3_. Let us discuss briefly the advantages and disadvantages of this approach. The advantage of this methodology (with respect to the three-phase method) is that the simulations reach equilibrium and one needs to simulate two phases (rather than three). From a computational point of view, it could be a little bit cheaper, as 500 ns is usually enough to reach equilibrium, whereas, in the three-phase method, one may need runs of the order of microseconds or more to clearly detect melting/growing of the methane hydrate. In addition to that, the three-phase method exhibits some degree of stochasticity, since some trajectories, especially at *T* close to *T*_3_, must usually be repeated using different initial seeds to ensure the hydrate phase grows or melts. These repetitions are unnecessary for the simulations for calculating solublities if the systems are well equilibrated. In any case, there is no free lunch and the method is also expensive. The efficiency of both routes is similar in terms of computer time, with the solubility route being slightly less demanding. Nevertheless, both routes are correct and should lead to consistent values of *T*_3_.

Notice that, from a thermodynamic point of view, above *T*_3_, there should be no hydrate, and the reason why we can observe it is because the nucleation of the gas phase is an activated process. On the other hand, below *T*_3_, one of the two phases (either water or methane depending on their relative amounts) should not exist. One should expect the nucleation of the hydrate, and then the growth of the hydrate until one of the two components (either water of methane) is totally consumed (see Figure 1 of ref (10)). The reason why we can evaluate the solubilities under metastable conditions^72^ is because the nucleation of either the hydrate or the gas is an activated process that does not take place in the time window (500 ns) required to determine the solubility with high accuracy. We confirmed that in the simulations used to determine the solubilities no nucleation of a hydrate was observed.

### III.D Revisiting *T*_3_ from Direct Coexistence Methods

**Figure 5:**
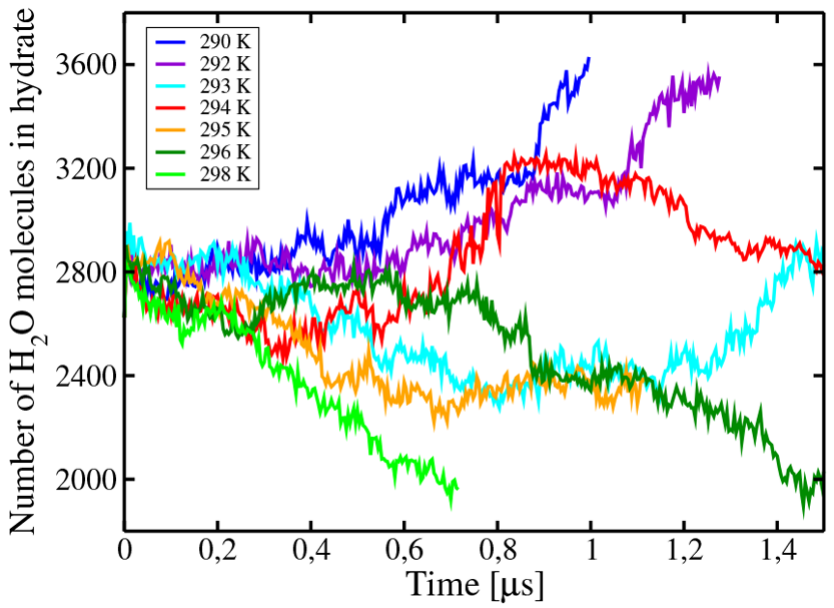
Time evolution of the size of the largest solid cluster at *p* = 400 bar for several temperatures as obtained from direct coexistence simulations of the hydrate–water–gas system. The hydrate melts clearly at 298 and 296 K. The hydrate grows at 290 and 292 K. Results at 293, 294, and 295 K do not show clear evidence of melting or growing, suggesting a triple point temperature *T*_3_ = 294(2) K at 400 bar. Notice that close to *T*_3_ the behavior is somewhat stochastic. In fact, at *T*_3_, the probability of melting or growing should be around 50%.

For the system considered in this work, Conde and Vega^10^ by using the three-phase direct coexistence method obtained *T*_3_ of 302 and 297 K, respectively, using two different system sizes and much shorter runs. It is of interest to revisit these calculations. For that purpose, we have used a three-phase system with the hydrate, water, and the gas phase. Interfaces were parallel to the *XY* plane. The system consisted of 5944 water molecules and 1512 methane molecules, and the size of the simulation box was equal to about 4.8 × 4.8 × 12.2 nm^3^. Simulations were carried out for a few temperatures in a range of 290–298 K. In order to maintain constant pressure, anisotropic scaling of the box was applied. The three-phase system was prepared in order to determine the temperature of coexistence of three phases at a pressure of 400 bar. In order to accomplish that, the changes in time of the amount of the solid phase in the system at different temperatures were evaluated, with the use of the  order parameter (see section S1 of the Supporting Information for additional information). The temperature of the three-phase coexistence was determined as an average of the lowest temperature at which the melting of the solid phase was observed and the highest temperature at which the hydrate was seen to grow. Results for the evolution of the number of solid molecules are presented in Figure 5. For temperatures above 296 K, the hydrate phase melts, whereas, for temperatures below 292 K, it grows. For the temperatures 293, 294, and 295 K, the system with the three phases remains stable even after runs of the order of a microsecond. As can be seen, the results of this work suggest 294(2) K as the value of *T*_3_ for this system from direct coexistence simulations, which is consistent with the value 297(2) K obtained from the solubility calculations using a cutoff of 9 Å or the value of 295 K using a cutoff of 17 Å. With all of these results, we recommend 295(2) K as the temperature of *T*_3_ for this system.

In Table 2, the values of *T*_3_ reported by various authors for the same model TIP4P/Ice and a LJ center for methane are presented. As can be seen, there is some scatter, some of which results from different values of cutoffs used in the simulations. The two calculations of this work are in agreement with the error bar. Taking all factors into consideration, our current estimated value is 295(2) K. This value (with its error bar) is consistent with a number of previous studies^14,16−18,73^ (some studies provide a slightly higher value and others slightly lower), although certainly lower than the value reported by Jensen et al.^15^ and lower than one of the values reported by Conde and Vega.^10^

**Table 2:** Values of *T*_3_ at *p* = 400 bar for the Model Considered in This Work (TIP4P/Ice and a LJ Center for Methane) as Obtained in This Work from Two Different Routes and as Obtained from Other Authors^a^

*T*_3_ (K)	ref.	cut-off used
302(3)	Conde and Vega^10^	0.9 nm
314(7)	Jensen et al.^15^	1.0 nm
297(8)	Conde and Vega^10^	0.9 nm
293.4(9)	Michalis et al.^14^	1.1 nm
293.5(5)	Fernández-Fernández et al.^16^	1.1 nm
290.5(5)	Fernández-Fernández et al.^16^	1.1 nm
297.8	Waage et al.^18^	1.0 nm
297(2)	solubility calculations—this work	0.9 nm
295(2)	solubility calculations—this work	1.7 nm
294(2)	direct coexistence—this work	0.9 nm
295(2)	recommended value—this work	

a All results of the table were obtained from the direct coexistence method, whereas those of Waage et al. were obtained from free energy calculations (we interpolated the results for 100 and 500 bar from these authors). The first two values are not compatible with the results of this work (even considering the combined error bars).

The value of this work is also in good agreement with the experimental value of *T*_3_ at 400 bar, which is equal to 297 K.^1,74^ As was shown before,^75^ water models with melting points of ice close to the experimental value give good values of *T*_3_, which is further confirmed in this work.

### III.E Curvature Effects on the Solubility of Methane from the Gas Phase

In section III.A, we have evaluated the solubility of methane in water from the gas phase when there is a planar interface between the aqueous solution and the gas phase. However, one could have a curved interface between the two phases and that could modify the value of the solubility. To analyze this, we have prepared bubbles of different sizes and inserted them into an aqueous phase having around 5000 molecules of water (the number of molecules of methane was different depending on the size of the bubble and the thermodynamic conditions but was in the range of 400–700). The simulation box was cubic, and we used isotropic *NpT* simulations. The system consists of a bubble of methane in the center of the simulation box (notice that the bubble is subject to Brownian motion and it can certainly move) surrounded by water. Radial density profiles of methane around the center of mass of the bubble were computed. The position of the center of mass of the bubble was evaluated separately in every frame of the trajectory based on the locations of the density maxima of methane in the *X*, *Y*, and *Z* directions, as was done in our previous work with LJ bubbles.^76,77^

Simulations were performed for 500 ns, and averages were obtained over the last 250 ns. Notice that the bubbles were stable all along the simulation. Once the system reaches equilibrium, it is possible to compute the solubility of methane for this spherical (gas–water) interface. Notice that when the system reaches equilibrium the chemical potentials of methane and water are identical in the two phases, and the same is true for all temperatures. However, pressure is now an inhomogeneous property and is different in the two phases. The condition of chemical equilibrium for this spherical interface is now written as
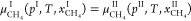
9
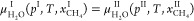
10

For convenience, when having a spherical interface, the aqueous solution will be denoted as phase I, whereas the gas phase forming the bubble will be denoted as phase II. Before continuing, it is important to clarify some aspects about the pressure in inhomogeneous systems. It is possible to define locally (at each point in space) a pressure tensor using, for instance, the Irving–Kirkwood (IK) criteria or that proposed by Harasima (H) (there is no unique way of locally defining the pressure tensor).^65^ In a homogeneous system, the pressure tensor is identical in all points of the sample. However, this is not the case in an inhomogeneous system. It is interesting to point out that the average value of a certain component of the pressure tensor in the entire system does not depend on the choice (IK or H) used to define it locally. The second interesting point is that for a system with a spherical interface (i.e., bubble) the average of the trace of the pressure tensor in the entire system is identical to the pressure of the external phase (see the Appendix of ref (78) for proof of this, which follows from the condition of mechanical equilibrium^79^ that states that the divergence of the pressure tensor should be zero ∇·*p* = **0**). In short, in an inhomogeneous system, the pressure of the system (i.e., the average of the trace of the pressure tensor) is identical to the pressure of the external phase (i.e., the aqueous solution in this case) and corresponds to the pressure applied by the isotropic barostat.

**Figure 6:**
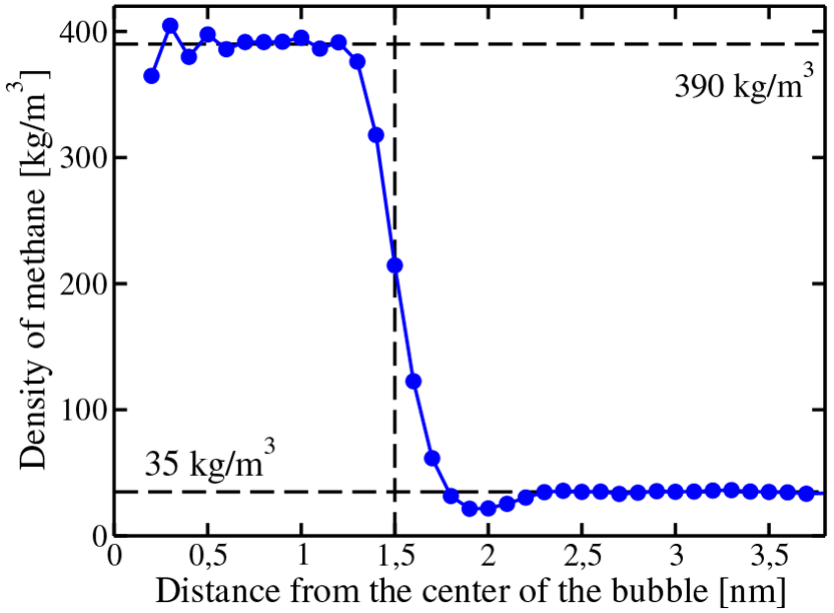
Density profile at 260 K of a bubble with radius 1.50 nm. The vertical line is the radius of the bubble as obtained from the equidensity criteria (i.e., when the density of methane is simply the average of the values inside and outside the bubble).

In Figure 6, the density profile of methane is shown (in units of mass per unit of volume) as a function of the distance from the center of the bubble (denoted as *R*). As can be seen, the bubble has a certain size which will be assigned as *R*_bubble_. There is not a unique criteria^65^ to define the radius of a bubble (we shall discuss this issue in detail later). For the time being, we shall use a simple criterion. We shall determine the radius of the bubble *R*_bubble_ as the value of *R* at which the density of methane in the density profile is just the arithmetic average of the density of methane in water and in the bubble. In previous work^76,77^ dealing with bubbles within a liquid for the Lennard-Jones system, we denoted this radius as the equidensity radius *R*_eq_. Mathematically, *R*_eq_ is defined as
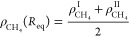
11where ρ_CH_4__ is the density of methane, either in phase I or in phase II (right-hand side) or in the density profile (left-hand side).

In Table 3, the solubilities of methane for bubbles of different sizes are shown. Results are also included in Figure 4. As can be seen, reducing the size of the bubble at constant temperature and global pressure increases the solubility of methane. At 290 K, a bubble of around 3 nm increases the solubility by a factor of 3 with respect to the planar interface. At 260 K, bubbles between 1.5 and 1.35 nm increase the solubility of methane by a factor of 4–5 with respect to the planar interface. This is in agreement with results of previous work.^25^

**Table 3:** Solubilities of Methane in the Aqueous Phase When in Contact with Bubbles of Radius *R*_bubble_^a^

*T* (K)	*R*_bubble_ (nm)	*x*_CH_4__	*x*_CH_4__(*R*)/*x*_CH_4__(∞)
250			
	1.77(3)	0.0553(28)	4.5
	∞	0.0123(46)	
260			
	1.35(1)	0.0464(9)	5.2
	1.49(2)	0.0419(14)	4.7
	1.98(1)	0.0303(13)	3.5
	∞	0.0089(3)	
290			
	1.78(1)	0.0137(7)	3.2
	2.12(1)	0.0116(2)	2.7
	∞	0.0043(7)	

a The value infinity for the radius indicates a planar interface. Radii are given in nm. Numbers in parenthesis indicates the uncertainty of the results.

The increase in the solubility of methane in the presence of bubbles with respect to the planar interface at constant temperature can be explained easily by noting that the local pressure inside the bubbles is not 400 bar (i.e., the pressure of the external phase) but higher, as can be understood from the Laplace equation. Higher pressures mean higher chemical potentials, and assuming ideal behavior for methane in water, that also means higher molar fraction. Let us try to understand the values obtained of the solubility on a theoretical basis. Let us assume that the chemical potential of methane in the water phase (phase I in our case) can be described as
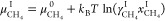
12where μ_CH_4__^0^ is just the standard state of methane in water which depends only on *T* and *p* but not on composition. By assuming ideal behavior (which seems reasonable taking into account the low solubility of methane in water), the change in the chemical potential of methane in the aqueous phase when in contact with a spherical bubble and with a planar interface can be estimated approximately (i.e., neglecting changes in the activity coefficient) as
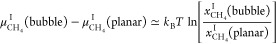
13

Since the solubility can change by a factor of 5, the chemical potential of methane changes significantly when in the presence of bubbles (around 1.6 in *k*_B_*T* units). Estimates of the chemical potential obtained from this route are shown in Table 4.

**Table 4:** Radius of the Bubble (as Given by *R*_eq_) and Chemical Potential of Methane in the Bubble (with Respect to the Chemical Potential of Bulk Methane at 400 bar)^a^

*T* (K)	*R*_bubble_ (nm)	Δμ_CH_4__ (*k*_B_*T*) (eq 13)	*p*^II,μ^ (bar)	σ_s_ (mJ/m^2^)
250	1.77(3)	1.50(6)	1062(25)	58.59
260	1.35(1)	1.65(2)	1147(9)	50.42
	1.49(2)	1.56(2)	1103(8)	52.37
	1.98(1)	1.22(4)	936(19)	53.06
290	1.78(1)	1.16(5)	859(21)	40.85
	2.12(1)	0.98(2)	785(5)	40.81

a Thermodynamic pressure inside the bubble (i.e., the value of the pressure of a bulk phase of methane having the same chemical potential as that found in the bubble). Interfacial free energy between the bubble and the aqueous phase (in mJ/m^2^) as estimated from the Laplace equation assuming the equi-density radius *R*_eq_ is the radius at the surface of tension^65,80^*R*_s_.

It is interesting to determine what would be the pressure of a bulk phase of methane needed to have the same chemical potential as methane in the external phase. We shall call that the thermodynamic pressure *p*^II,μ^ of phase II. This pressure can be obtained easily by integrating (along an isoterm) the volume per molecule of pure methane
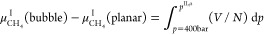
14where the integrand (*V*/*N*) is the volume per molecule of a bulk phase of methane at pressure *p*, which can be obtained easily from simulations of pure bulk methane in the *NpT* ensemble. Notice that this thermodynamic pressure *p*^II,μ^ is the one that enters into the thermodynamic description of the internal phase in Gibbsian formalism (in section S2 of the Supporting Information, we discuss this issue further). Results for this thermodynamic pressure are presented in Table 4.

Finally, we can estimate a value of σ_s_ for the bubble–water interface using the Laplace equation
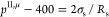
15where *R*_s_ is the radius at the surface of tension^80^ and σ_s_ is the interfacial free energy at the surface of tension (see refs (65, 80), and (81) for a detailed description of all of the subtle issues concerning the thermodynamics of curved interfaces and for proof that the Laplace equation only holds when the dividing surface is located at the radius of tension). In general, the value of *R*_s_ is unknown, as its determination requires free energy calculations. However, if one assumes that
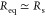
16then one can obtain values of σ_s_. They are shown in Table 4. As can be seen, the value of σ_s_ is different from that of a planar interface, as also found in other systems.^82,83^ We found that the values of σ_s_ for the bubbles of this work are around 10–20% lower than those of the planar interface. That indicates a positive Tolman length for the methane–water interface.^83^ A similar decrease in the value of the surface tension for small LJ droplets was found by Vrabec et al.^84^

One may wonder if it would be possible to increase the solubility of methane beyond a factor of 5–6 with respect to the value of the planar interface by making the bubbles even smaller. We have attempted to do that by removing particles of methane from the bubbles to make them smaller. However, unfortunately at around 1.25 nm, they become mechanically unstable and disappear by dissolving into the water phase. Thus, it is not possible to increase the solubility of methane with respect to that of the planar phase up to an arbitrary limit. It seems it is only possible to increase the solubility by a factor of 4–6 by using bubbles slightly above the nanometer size.

### III.F Curvature Effects on the Solubility of Methane from the Hydrate Phase

**Figure 7:**
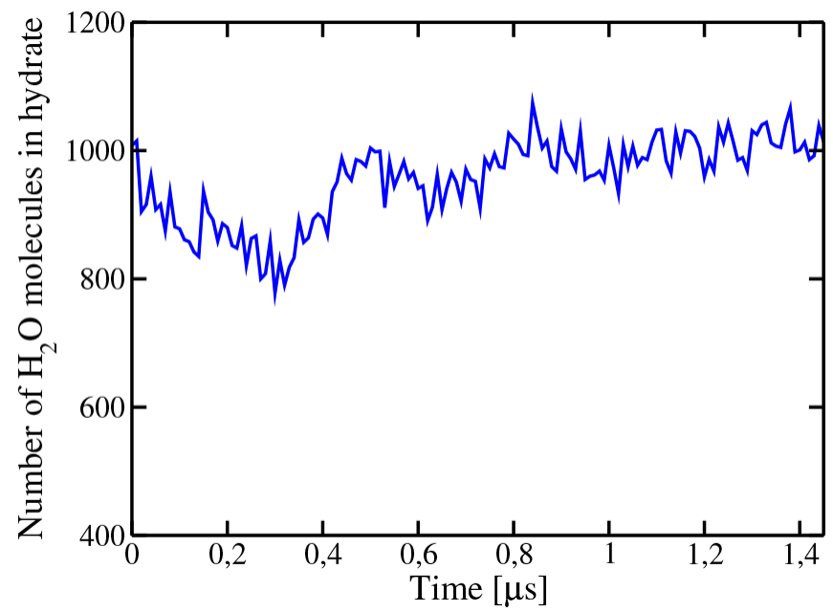
Time evolution of the size of the spherical solid cluster of hydrate at 250 K and 400 bar, for the system consisting of 4786 molecules of water and 200 molecules of methane. As can be seen, the cluster is stable and neither grows nor melts. The concentration of methane in the aqueous solution is presented as a light blue filled square at 250 K in Figure 4.

It seems of interest to now compute the solubility of methane in water when in contact with the hydrate but now with a spherical interface. For that purpose, we shall insert a spherical solid cluster of the hydrate solid phase into a cubic box of water with some methane previously dissolved. It was observed that for certain concentrations of methane in solution the solid cluster was fairly stable. In fact, after running over 1 μs, the size of the cluster did not change much and fluctuated around an average value. To determine the size of the cluster, we used the order parameter proposed by Lechner and Dellago.^56^ In particular, we used . Details are described in the Supporting Information. The time evolution of the size of the cluster is shown in Figure 7. As can be seen, the cluster remains stable. The concentration of methane in the external phase was constant, and its value was 0.0046 (in molar fraction), which is about 5 times higher than the solubility from the hydrate when the interface is planar.

The physics is quite similar to that found for bubbles. The presence of curvature increases the solubility of methane from the hydrate. One can understand this again by regarding the Laplace equation and understanding that the spherical solid cluster is at higher pressure, therefore the chemical potential of the methane increases and so does the solubility. A similar effect was found in a previous study determining the solubility of NaCl in water or that of a LJ solid into the fluid for both planar and curved interfaces.^85^ The solubility of the spherical solid was higher than that of the planar interface. It is nice to know that the solubility increases with the presence of curvature, but one should be aware that to determine the solubility of a certain force field one should always use the planar interface.

### III.G Stability of Inhomogeneity in a Finite Size System

It is well-known that for one-component systems it is possible in certain ensembles (for instance, *NVT*) to have a stable inhomogeneous system.^86^ Depending on the values of *N*, *V*, and *T*, one can find inhomogeneous systems with spherical, cylindrical, or planar interfaces as the most stable ones (i.e., at the absolute minimum of the Helmholtz free energy *F*).^76,77,86,87^ One can even have local minima in *F* which are metastable with respect to others (e.g., a system with a spherical interface which is metastable with respect to a cylindrical or planar interface).^88^ These local minima can be separated by free energy barriers so that one can stay in these minima for a long time. However, for pure components, as soon as one switches to the *NpT* ensemble, the inhomogeneous system is unstable^87,89^ and it will evolve toward one of the two phases at equilibrium (regardless of whether one has a spherical, cylindrical, or planar interface). The summary is that in pure components the existence of a stable (or metastable) inhomogeneous system is possible in the *NVT* ensemble (see ref (88) for a discussion of whether this is also possible for other ensembles).

What is the situation for mixtures? A finite size mixture is defined by four variables. For instance, one could use *N*_1_, *N*_2_, *p*, and *T* as in our *NpT* simulations. The main difference between a mixture and a pure component system is that now an inhomogeneous system (with a planar or curved interface) can be at equilibrium (either stable or metastable) in the *N*_1_*N*_2_*pT* ensemble. Thus, the inhomogeneous system will be stable/metastable, as it would live in global/local minima of the Gibbs free energy *G*. Obviously, the inhomogeneous system will be unstable if one changes to the semigrand ensemble^90^ where *N*_1_, μ_2_, *p*, and *T* are constant as it will evolve toward one of the two phases at equilibrium. Thus, mixtures in the *N*_1_, *N*_2_, *p*, *T* ensemble follow the behavior of pure component systems in the *NVT* ensemble and mixtures in the *N*_1_, μ_2_, *p*, *T* ensemble follow the behavior of pure component systems in the *NpT* ensemble.

The message is that for a certain value of *N*_1_, *N*_2_, *p*, and *T* one can have local minima of *G* describing either a homogeneous system or several inhomogeneous systems with spherical, cylindrical, or planar interfaces. In general, there will be free energy barriers separating these states. If the free energy barriers are large, the system can stay for quite long times in these configurations. If the free energy barriers are small, the system will overcome these free energy barriers and will evolve to the global minima in *G*. It goes beyond this study to analyze if the curved interfaces considered so far (for instance, that of the spherical solid cluster of hydrate in equilibrium with the solution shown in Figure 7) correspond to the absolute minima in *G* for the selected values of *N*_1_, *N*_2_, *p*, and *T*. It is clear that if they are stable for quite long times (i.e., larger than 500 ns) they correspond to local minima in *G*.

Now we shall analyze in detail the thermodynamic driving force for the formation of the solid hydrate.

### III.H Driving Force for Nucleation of Hydrates

Below *T*_3_, one should not have water in contact with methane (regardless of whether one has planar or curved interfaces). One should have the hydrate in contact with either water or the gas. If the ratio *N*_H_2_O_/*N*_CH_4__ > 5.75, one should have a hydrate–water system. If the ratio is smaller, one should have a hydrate–gas phase system. The formation of the hydrate in the aqueous phase can be written as a chemical reaction (see Kashchiev and Firoozabadi^22^) occurring at constant *p* and *T*:
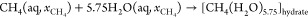
17It is useful to treat the hydrate as a new compound which has one molecule of methane and 5.75 of water and to define a chemical potential for the hydrate. Obviously the chemical potential of the hydrate is just the sum of the chemical potential of methane in the solid plus 5.75 times the chemical potential of water in the solid. If one is interested in changes in the stoichiometry of the hydrate, then one should refer to the chemical potential of the individual components of the solid. However, the occupancy of methane cages is large (usually well above 90%) and we shall assume here full occupancy (further work is needed to understand if the non-stoichiometry is due to either thermodynamics or kinetic reasons). We shall assume here that all cages of the hydrate are occupied by methane. Therefore, we shall refer to the chemical potential of the hydrate (and not to the chemical potential of its individual species). This is analogous to the case of NaCl in the solid phase. One could refer to the chemical potential of Na and Cl individually, but it is more useful to evaluate the chemical potential of NaCl in the solid phase (which of course is just the sum of the chemical potentials of the individual ions). Therefore, the compound [CH_4_(H_2_O)_5.75_]_solid_ will be denoted simply as the “hydrate” and one molecule of the hydrate means one molecule of [CH_4_(H_2_O)_5.75_] in the solid.

What is the value of the driving force for nucleation of the hydrate from the solution? We shall denote it as *Δμ*_nucleation_, and it is given by^22,23^
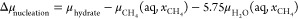
18where it should be understood that each individual chemical potential is computed at the same *p* and *T*. Obviously the value of *Δμ*_nucleation_ depends also on the value of the variable *x*_CH_4__. There is a particular value of *x*_CH_4__ of great interest from a practical point of view. It corresponds to the case in which the value of *x*_CH_4__ is given by the solubility of methane from the gas phase (unless otherwise stated, via a planar interface). This is the way experiments on the nucleation of hydrate are performed (i.e., a water phase in contact via a planar interface with the gas phase of methane). We shall denote this value simply as *Δμ*_nucleation_^EC^ (where the superindex EC indicates experimental conditions). Notice that in this case the value of *x*_CH_4__ is not an independent variable, as it is entirely determined by *p* and *T*. The chemical potential of the hydrate does not depend on composition and is also entirely determined by *p* and *T*. Unfortunately, very little is known on the experimental values of *Δμ*_nucleation_^EC^. Kashchiev and Firoozabadi used experimental information to roughly evaluate its magnitude.^22,23^ It seems of interest to determine it for the force field used in this work. It could be useful for future studies of nucleation using the same force field. It can also provide clear trends of the experimental values. However, force fields are approximate so that the obtained values will not be identical to those found in experiments.

#### III.H.1 Route 1 to *Δμ*_nucleation_^EC^

Let us estimate *Δμ*_nucleation_^EC^ which by definition is *Δμ*_nucleation_ when the molar fraction of methane is given by the solubility of methane from the gas phase via a planar interface. We shall illustrate how to compute it along the *p* = 400 bar isobar. The first route to *Δμ*_nucleation_^EC^ is simple: one will evaluate each of the terms of eq 18 individually, and then, one will compute *Δμ*_nucleation_^EC^. When evaluated in this way, it will be denoted as *Δμ*_nucleation_^EC1^. At *T*_3_ for 400 bar, we know that *Δμ*_nucleation_^EC^ = 0 (i.e., the chemical potentials of methane and water in the solution and in the hydrate are identical), although we do not know the individual values of μ_CH_4__ and μ_H_2_O_. However, this is not a problem, as we are interested in changes in chemical potentials and not in individual values. For this reason, we shall set to zero the chemical potential of methane, water, and hydrate at *T*_3_ (i.e., 295 K).

To estimate μ_CH_4__(aq, *x*_CH_4__), we shall use eq 6 which is almost exact, as the solubility of water in the methane gas phase is negligible. The calculation of μ_hydrate_ is both simple and exact and is given by
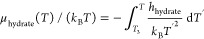
19where *h*_hydrate_ is the enthalpy of one “molecule” of the hydrate (i.e., the CH_4_(H_2_O)_5.75_).

To evaluate *Δμ*_nucleation_^EC^, we also need to evaluate μ_H_2_O_(aq, *p*, *T*, *x*_CH_4__). Let us write the expression of the chemical potential of water in the solution at *T*_3_ and at *T*
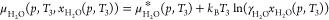
20where for simplicity we have omitted the dependence of γ with *T*, *p*, and the composition. Since the solution is diluted, one may approximate the value of γ_H_2_O_ to 1 to obtain
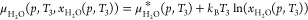
21

Let us now write the same expression at *T*:
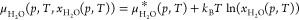
22

Therefore, the change in chemical potential along the isobar is obtained as
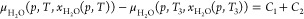
23
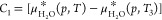
24
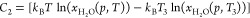
25

As can be seen, the chemical potential has two contributions (*C*_1_ and *C*_2_). The first one accounts for changes in the chemical potential of pure water due to the temperature and the second one for changes in the molar fraction of water. Since the standard state for water is pure water, one simply obtains (*h*_H_2_O_ being the enthalpy per particle of pure liquid water)
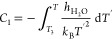
26

**Figure 8:**
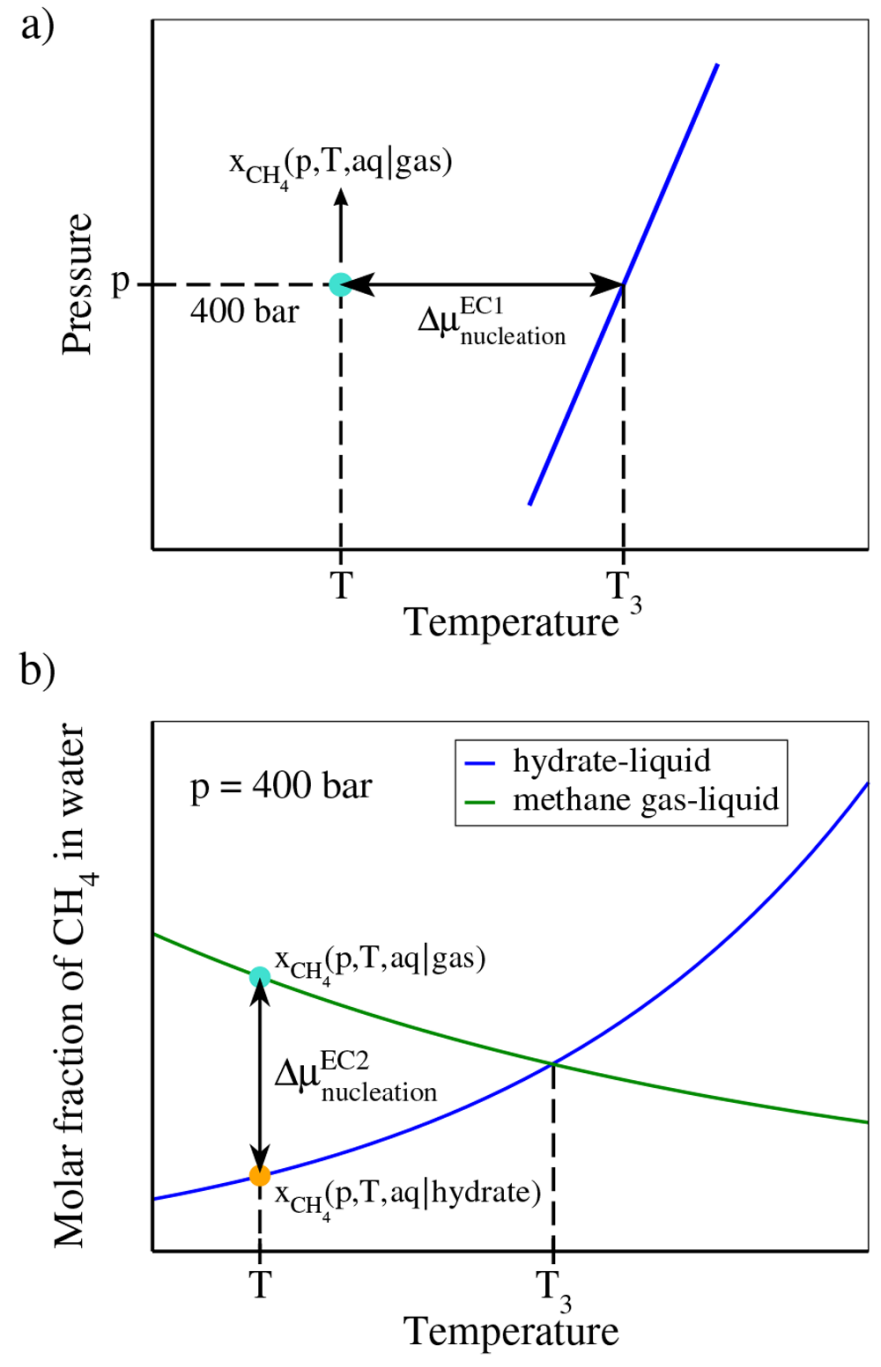
Schematic depiction of two routes for obtaining *Δμ*_nucleation_^EC^. The points represent the equilibrium concentrations of methane in water in gas–liquid (light blue points) and hydrate–liquid (orange point) systems with a planar interface at temperature *T*. (a) Route 1 to *Δμ*_nucleation_^EC^. (b) Route 2 to *Δμ*_nucleation_^EC^.

The second contribution is quite small and can be either neglected or estimated from the solubility of methane in water. Notice that the change in chemical potential is also the chemical potential of water at *T*, as the chemical potential of water was set to zero at *T*_3_. The route of obtaining *Δμ*_nucleation_^EC1^ is schematically depicted in Figure 8a.

As has been discussed above to evaluate *Δμ*_nucleation_^EC1^ simulations of pure methane, pure water and pure hydrate are needed. In the case of pure methane and pure water, a cubic system having 1000 molecules will be used. In the case of the pure hydrate, a system having 1242 molecules of water and 216 molecules of methane will be used. Isotropic *NpT* simulations will be performed for pure water and pure methane. For the hydrate, we used anisotropic *NpT* scaling (although as the solid has cubic structure isotropic scaling would have also been possible). Systems were simulated at a few temperatures in the range 250–310 K for 100 ns.

**Figure 9:**
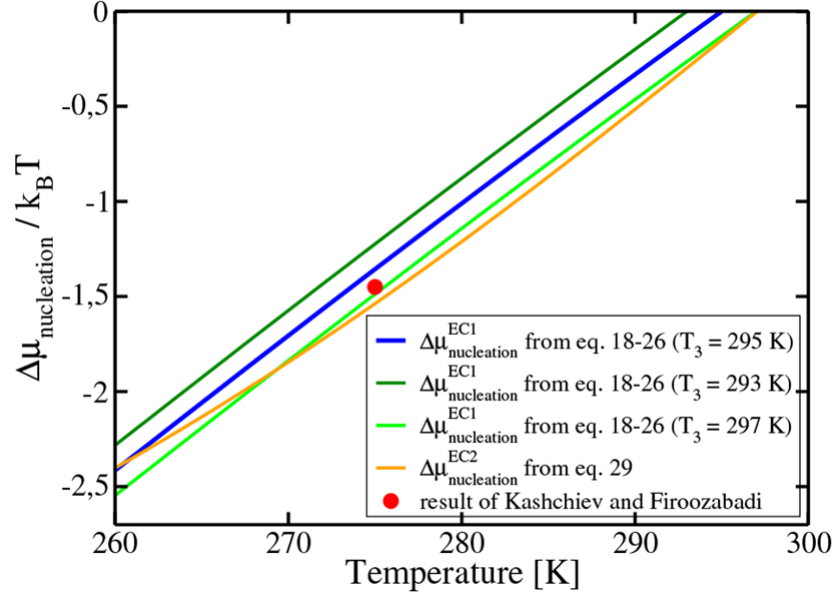
Chemical potential for hydrate formation *Δμ*_nucleation_^EC1^ and *Δμ*_nucleation_^EC2^ as a function of temperature along the 400 bar isobar. For the first route, we use 295 K as the value for *T*_3_. Obviously, at this temperature, *Δμ*_nucleation_ is zero, as the three phases are in equilibrium. Results assuming *T*_3_ = 293 K and *T*_3_ = 297 K are also shown. The results include the small correction due to the change in the molar fraction of water with temperature (eq 25). To obtain the values of *Δμ*_nucleation_^EC2^, we fitted the solubility values (from runs with a cutoff of 9 Å) for temperatures in the range of 260–300 K to curves, which are given by *x*_CH_4__ = e^–0.0332·*T*+3.744^ + 0.00135 for the gas–liquid system (*x*_CH_4__ = 0.0009 + exp(−0.0294 * *T* + 2.634) when the cutoff is 17 Å) and *x*_CH_4__ = e^+0.0622·*T*−24.268^ + 0.0005 for the hydrate–liquid system. Values of Δ*μ*_nucleation_^EC2^ were determined using the runs with a cutoff of 9 Å, leading to *T*_3_ = 297 K (for this reason, the agreement is better with *Δμ*_nucleation_^EC1^ obtained for this choice of *T*_3_). For comparison we have also included (red point) the value of *Δμ*_nucleation_ estimated from experimental results by Kashchiev and Firoozabadi^22,23^ at 194 bar and for a supercooling of 20 K with respect to *T*_3_ at this pressure.

Values of *Δμ*_nucleation_^EC1^ are presented in Figure 9. As can be seen for a supercooling of about 35 K (i.e., 260 K), it amounts to around 2.4 in *k*_B_*T* units. Using experimental results, Kashchiev and Firoozabadi^22^ estimated a value of *Δμ*_nucleation_^EC^ of about 1.5 *k*_B_*T* at a pressure of 194 bar for a supercooling of 20 K (see Figure 4 of ref (22)). We have included this result in Figure 9, and as it can be seen, our results are consistent with those of Kashchiev and Firoozabadi (although our values are for the force field of this work and those of Kashchiev and Firoozabadi for experiments and in addition both results were obtained at different pressures). Notice that to evaluate *Δμ*_nucleation_^EC^ Kashchiev and Firoozabadi needed to estimate a number of properties for the hydrate and for the pure components.

In Table 5, the individual contributions to *Δμ*_nucleation_^EC1^ are provided. It is clear that the major contribution is made by the hydrate and water. The methane contributes in a much smaller magnitude to *Δμ*_nucleation_^EC1^.

**Table 5:** Chemical Potential Change (in *k*_B_*T* units) of Hydrate Formation under Experimental Conditions (i.e., *x*_CH_4__ Given by the Gas–Liquid Equilibrium with a Flat Interface) Evaluted from the First Route *Δμ*_nucleation_^EC1^ at 400 bar for Several Temperatures^a^

*T* (K)	Δμ(CH_4_(aq))	5.75× Δμ(H_2_O(aq))	Δμ(hydrate)	Δμ_nucleation_^EC1^
250	–0.13639	–23.34586	–26.62722	–3.14497
260	–0.09386	–17.33869	–19.85317	–2.42062
270	–0.05914	–11.84815	–13.61482	–1.70753
280	–0.03118	–6.81213	–7.85394	–1.01062
290	–0.00909	–2.17910	–2.52036	–0.33217
295	0.0	0.0	0.0	0.0

a At *T*_3_, i.e., 295 K, the chemical potential of all species was set to zero.

Let us finish this section by presenting a quite simple (but approximate) route to determine *Δμ*_nucleation_^EC^ by using the dissociation enthalpy of the hydrate. The dissociation enthalpy *h*_diss_ is defined as the enthalpy change of the process:
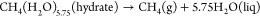
27

When calculating dissociation enthalpies, one assumes that the hydrate dissociates into pure water and pure methane. Of course, although this is the definition of the enthalpy of dissociation, one should keep in mind that when the actual hydrate dissociates there will always be a small amount of methane dissolved in water and an even smaller amount of water dissolved in the methane gas phase. The dissociation enthalpy of the model used in this work is obtained simply by performing simulations of the pure phases (hydrate, water, and methane) at several temperatures along the isobar. Dissociation enthalpies obtained from simulations are reported in Table 6.

**Table 6:** Dissociation Enthalpy (per mol of Methane) of the Hydrate at Several Temperatures at 400 bar Obtained from Computer Simulations of the Force Field Considered in This Work

*T* (K)	Δ*h*_diss_ (*k*_B_*T*)	Δ*h*_diss_ (kJ/mol)
250	18.53	38.51
260	18.94	40.92
270	19.26	43.23
280	19.38	45.11
290	19.59	47.22
295	19.62	48.12
300	19.68	49.07
310	19.72	50.81

The values obtained are in good agreement with previously reported values for a similar system.^98^ Notice that one can obtain a simple (but approximate) expression to estimate the value of *Δμ*_nucleation_^EC^ by assuming that the enthalpy of dissociation does not change with the temperature and can be taken from its value at *T*_3_ and by neglecting the change in composition with temperature of the aqueous solution containing methane, thus obtaining
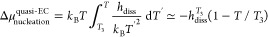
28

**Figure 10:**
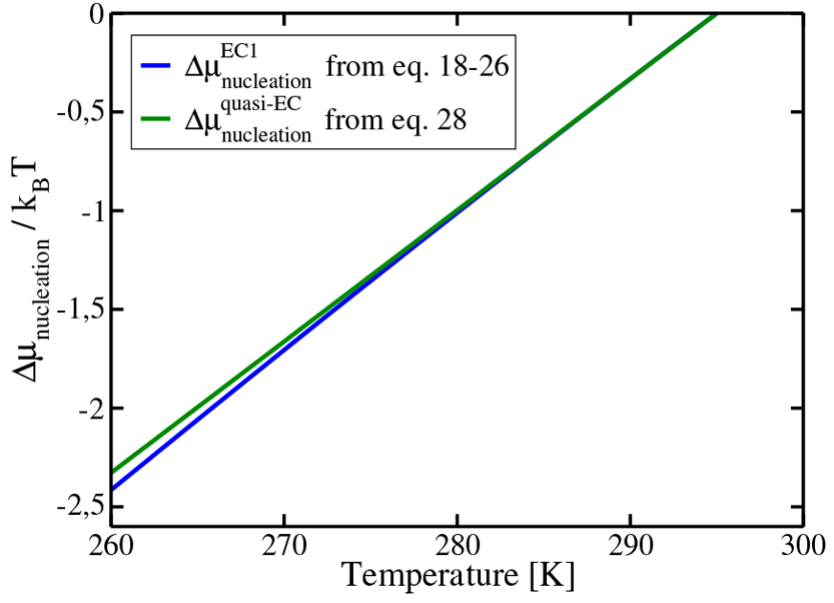
Values of *Δμ*_nucleation_^EC^ as obtained from assuming constant enthalpy of dissociation (eq 28) as compared to the more elaborated eq 18. We used the value 295 K for *T*_3_ at 400 bar.

The values obtained from this route are included in Figure 10. The agreement with the more elaborate expressions of eqs 18–26 is good, although it starts to deviate at large supercoolings.

Let us now present another possible route to estimate *Δμ*_nucleation_.

#### III.H.2 Route 2: Obtaining *Δμ*_nucleation_^EC^ from Values of the Solubility of Methane

In the previous route, we arrived to the state of interest with *p*, *T*, and *x*_CH_4__ = *x*_CH_4__(aq, *p*, *T*|gas) along an isobar starting from a point where the chemical potential of water and methane was identical in the solution and in the hydrate. However, it is also possible to arrive to the state *p* and *T* and composition *x*_CH_4__ arriving from another state with the same *p* and *T* but with different composition and where the chemical potentials of methane and water are identical in the solution and in the hydrate. In fact, along the solubility curve of methane from the hydrate, this condition is satisfied so that *Δμ*_nucleation_^EC^ = 0. Therefore, we just need to compute the change in chemical potential with composition at constant *p* and *T*. Notice that standard states depend only on *p* and *T* and not on composition, and since the hydrate chemical potential does not change when changing the composition of the solution, one obtains a new route (labeled with the superindex 2) to determine *Δμ*_nucleation_^EC2^
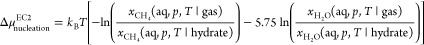
29where the vertical lines represent flat interface equilibrium with another phase (i.e., gas methane or the solid hydrate). This route for obtaining *Δμ*_nucleation_^EC2^ is depicted in Figure 8b. The negative sign arises from the fact that we have defined *Δμ*_nucleation_^EC^ in the direction of freezing so that one subtracts the chemical potential of methane and water in the aqueous solution. Since the solubility of methane is quite small, the molar fraction of water is close to 1 and one can neglect the second term on the right-hand side. This was done by Molinero and co-workers^8^ in previous work.

The values of *Δμ*_nucleation_^EC2^ obtained from this second route are shown in Figure 9, and they are compared to the ones of the first route. The agreement is quite good. Notice, however, that the shape of the curves obtained by EC1 and EC2 routes is slightly different (a curvature of the line obtained by the EC2 route can be seen). The reason for that is presumably related to the uncertainty of the values of solubilities used to calculate *Δμ*_nucleation_^EC2^—as can be seen in Figure 1 and Figure 3, there is some scatter in the results. Additionally, the solubility of methane in water when in contact with the hydrate is very low at low temperatures and even a small error may have a significant impact on the value of *Δμ*_nucleation_^EC2^.

In previous work, we have determined values of *Δμ*_nucleation_ for the formation of ice Ih (for a pure substance, the value of *Δμ*_nucleation_ is unique, as one does not need to specify the composition of the aqueous phase). For instance, for a supercooling of 35 K, we found a value of 0.29 in *k*_B_*T* units.^91^ At the same supercooling, the chemical potential change per unit of hydrate molecule is around 2.30 *k*_B_*T*, which after being divided by 5.75 yields a value of 0.40 *k*_B_*T* per molecule of water. The summary is that the driving force for the nucleation of the methane hydrate (at 400 bar) is significantly higher than that of the nucleation of ice (at 1 bar) when compared at the same supercooling. Other things being equal (i.e., interfacial energies), that means that nucleation of the hydrate should be more favorable than that of ice Ih.^92^

### III.I Helping Hydrate Nucleation

In the previous section, we have shown how to compute *Δμ*_nucleation_ for hydrate nucleation under “experimental conditions”, i.e., *Δμ*_nucleation_^EC^. By experimental conditions, we mean that the concentration of methane in water is that obtained at equilibrium for a planar water–gas interface at the considered values of *p* and *T*.

Would it be possible to help the nucleation of hydrate under “special conditions”? The answer to this question is positive. In fact, for certain values of *p* and *T*, by using brute force simulation, Sum and co-workers^25^ were able to nucleate the hydrate in less than a microsecond (provided that the gas phase was forming a bubble and the concentration of methane in water was in the range *x*_CH_4__ = 0.02–0.04). Under the same conditions of *p* and *T*, nucleation never occurred for the planar interface even after running for several microseconds. Definitely the bubbles helped, and the reason is simple. The solubility of methane increases. We shall denote as *Δμ*_nucleation_^*^ (with an asterisk) values of the change in chemical potential for the formation of the hydrate (i.e., values of *Δμ*_nucleation_) obtained under conditions where the solubility of methane is higher than that obtained for a planar water–gas interface at the considered conditions of *p* and *T* (for example, when one has a bubble of methane and not a planar interface). For simplicity, we shall denote as *x*_CH_4__^eq^ and *x*_H_2_O_^eq^ the molar fractions of methane and water in the aqueous phase when in equilibrium with the gas phase via a planar interface at a certain value of *p* and *T*. We shall denote *x*_CH_4__^*^ and *x*_H_2_O_^*^ values of the molar fractions in the aqueous phase different from these. If the composition of the aqueous solution is changed but *p* and *T* are unchanged, then the chemical potential of the hydrate phase does not change. However, there will be a change in the chemical potential of water and methane. One then obtains
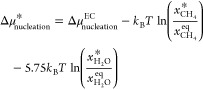
30

If we neglect the third term on the right-hand side (which is typically much smaller than the second one, as molar fractions of water in solution are typically above 0.95), then one obtains
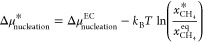
31

According to this expression, if the solubility of methane is increased by a factor of 3–5, then the value of *Δμ*_nucleation_^*^ becomes larger (in absolute value, as it is a negative number) by 1.1–1.6 *k*_B_*T* units with respect to *Δμ*_nucleation_^EC^. This is quite a significant increase. Around 20 K of additional supercooling would be needed to obtain a similar change in *Δμ*_nucleation_^EC^. Of course, 20 K of further supercooling slows down the dynamics. Therefore, bubbles help in the nucleation of the hydrate and would provoke a similar increase in the driving force as 20 K of further supercooling while keeping the dynamics fast. However, there is a limit in the help that bubbles can provide for brute force nucleation studies. As bubbles cannot be smaller than 1.25 nm (they are not mechanically stable for smaller sizes), the maximum increase that they can provide for nucleation is of around 1.6 *k*_B_*T* units. This is enough to force nucleation for moderate to high supercooling (i.e., in the range of *T* between 240 and 255 K). However, this is not enough to provoke spontaneous nucleation at small supercoolings (i.e., above 260 K). In fact, for pressures below 500 bar, Sum and co-workers^25,26^ were not able to nucleate hydrate at temperatures above 255 K even with bubbles.

**Figure 11:**
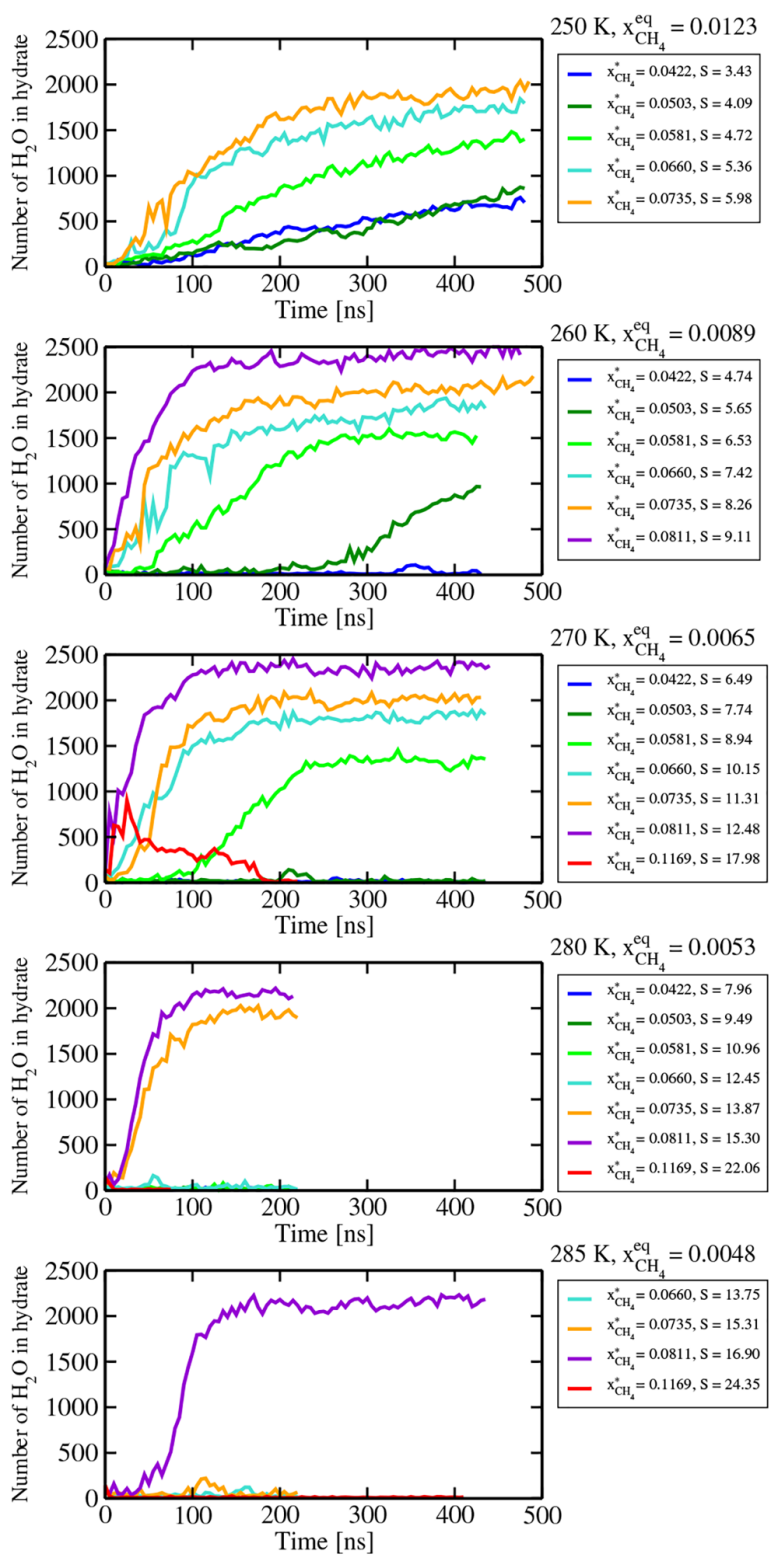
Size of the largest solid cluster of hydrate as obtained in brute force *NpT* simulations (*p* = 400 bar, cutoff = 9 Å) of homogeneous solutions at different temperatures and supersaturations. The value of the equilibrium solubility of methane (for a planar interface) at each temperature is shown. The actual values of the molar fraction of methane of each run are also shown, as well as the supersaturation *S* defined as the ratio *x*_CH_4__^*^/*x*_CH_4__^eq^. We obtained spontaneous crystallization for all temperatures up to 10 K below *T*_3_.

Would it be possible to nucleate the hydrate at even higher temperatures (i.e., above 260 K)? Bubbles are not enough, but extra help can be found. In fact, all that is needed is to have an “artificially” high value of *x*_CH_4__^*^. A possibility is to start from a highly supersaturated solution (i.e., one with a concentration many times higher than the solubility of the planar interface). The system would be now in a doubly metastable state. In fact, it will be metastable with respect to the formation of the hydrate, but it would also be metastable with respect to the formation of the gas phase (either in the form of bubbles, cylinders, or a planar interface). The phase that will appear first is the one with the lowest free energy barrier for nucleation. This was the approach used by Sarupria and Debenedetti,^93,94^ and it is also the path followed in pioneering studies on hydrate nucleation. We shall follow this approach. We will start from a homogeneous mixture having around 5000 molecules of water and the number of methane molecules required to have a certain molar fraction of methane. We typically used values of *x*_CH_4__^*^ in the range 0.042–0.117 (notice that 0.117 is only slightly below the composition of the hydrate which has a molar fraction of methane of around 0.15). Results of these runs are shown in Figure 11. As can be seen, we were able to observe the nucleation of the hydrate at all temperatures up to 285 K (i.e., just 10 K below *T*_3_!). These results are in line with the results obtained previously^95^ for a similar system at *p* = 500 bar—in this case, nucleation of hydrate was observed for temperatures in the range 250–285 K (up to 14 K below *T*_3_ for these pressure conditions).

At a temperature of 285 K, the supersaturation needed to observe the nucleation is almost 20 times the equilibrium solubility for the planar interface. Such a supersaturation provokes a shift in the chemical potential of methane of about 3 *k*_B_*T* units. Since *Δμ*_nucleation_^EC^ at this *T* is of about 0.7 *k*_B_*T*, it can be seen that a value of *Δμ*_nucleation_^*^ around 4 *k*_B_*T* is enough at this temperature to provoke nucleation in brute force runs. In general, values of *Δμ*_nucleation_^*^ higher than 4 *k*_B_*T* are enough to guarantee nucleation in brute force simulations. These values can be obtained below 255 K using bubbles or above this temperature using highly supersaturated homogeneous solutions.

It is worth pointing out that the hydrate nucleation is a transition from a disordered phase to an ordered phase with solid-like characteristics that are not necessarily crystalline. In our work, we were only interested in detecting the nucleation events, for which we used the  order parameter, while the details of the structure of the formed hydrate were not analyzed.

We tried to obtain nucleation even at higher temperatures (i.e., around 290 K) using high supersaturation. However, we were not successful. If the supersaturation was low, then there was no nucleation, and if the supersaturation was high, then the spontaneous formation of the bubbles of the gas phase took place and supersaturation was reduced to normal (i.e., 3–5 times), which is not enough to induce nucleation at these high temperatures close to *T*_3_. We also observed the formation of bubbles at lower temperatures (i.e., 270–285 K) but only for the highest concentration of methane considered—*x*_CH_4__ = 0.117. At 270 K, both the hydrate and the bubble were formed at the beginning of the simulation; however, the hydrate melted soon after. Although it is not an accurate method to determine *T*_3_, it is obvious that the highest temperature at which spontaneous formation of the hydrate was observed in a highly supersaturated solution provides a lower bound to a rough estimate of *T*_3_.

We must confess that we never succeed in forming ice Ih in brute force simulations of pure water using the TIP4P/Ice model. The fact that we are able to nucleate the hydrate in a few hundreds of ns while just 10 K below *T*_3_ is remarkable. The concentration of methane in water is the key to dramatically change the driving force for nucleation. This can be increased either by introducing bubbles or by starting from an homogeneous highly supersaturated solution.

## IV Conclusions

In this paper, we have studied at 400 bar the solubility of methane in water when in contact with either the gas or the hydrate with computer simulations using the TIP4P/Ice model for water and a simple LJ model to describe the methane molecule. The solubility of methane from the gas decreases as the temperature increases, revealing that the process is exothermic. The solubility of methane from the hydrate increases with temperature, showing that the process is endothermic. There is a temperature at which both curves intersect, and this determines the *T*_3_ value at which the three phases—gas, water, and hydrate—can remain at equilibrium at a certain pressure. Therefore, we show that a new method to evaluate *T*_3_ in hydrate research is available that requires performing simulations that reach equilibrium in all cases. Above *T*_3_, the hydrate is not thermodynamically stable. Below *T*_3_, one of the two fluid phases will not be thermodynamically stable. However, we observe metastability. Therefore, it is possible to have a water–gas interface at temperatures below *T*_3_, as the formation of hydrate is an activated process, and it is also possible to have the hydrate–water interface above *T*_3_, as the formation of the gas phase is an activated process. Since there is some scatter around the value of *T*_3_ for this system, we revisited also the direct coexistence method using larger systems than in our previous work. The final conclusion is that for this system *T*_3_ is located at 294(2) K from direct coexistence simulations and at 297(2) K from the solubility calculations (for a cutoff of 9 Å) and 295(2) K (for a cutoff of 17 Å). These values are lower than one of the values we reported some time ago (i.e., 302 K) and in better agreement with the estimates of other groups. With all of this information, we suggest 295(2) K as the *T*_3_ value for this system at a pressure of 400 bar.

We have analyzed the impact of a curved interface on the solubility of methane, concluding that the curvature increases the solubility. Both the solubility of methane from the gas and the solubility of methane from the hydrate increase due to curvature. The explanation is simple—the higher pressure inside the phase forming the sphere increases the chemical potential of methane and therefore increases the solubility (as can be understood by describing the behavior of methane in water by an ideal mixture model with activity coefficient equal to 1). We have estimated the change in the chemical potential of methane due to curvature. The fact that bubbles increase the solubility has been described previously. We show here that the smallest bubble that is mechanically stable has a radius of about 1.25 nm. Below this size, the bubbles collapse. Therefore, by using bubbles, one can increase the solubility of methane with respect to a planar interface by a maximum factor of 4–6. It is not possible to increase the solubility beyond this value. However, the fact that it is possible to have stable solid clusters of the hydrate in contact with the solution has not been reported before. The existence of spherical stable interfaces in the *NVT* ensemble for pure components has been described before for HS (solid–fluid) and LJ systems (both solid–fluid and fluid–fluid). For pure components, it is not possible to have a stable spherical interface in the *NpT* ensemble. Here we show that this is possible for mixtures and not only for the gas–water interface but also for the hydrate–water interface.

The driving force for nucleation is the change in chemical potential when forming the hydrate. There are almost no estimates of its value with the exception of the work of Kashchiev and Firoozabadi.^22,23^ Even from experiments, it is difficult to estimate this magnitude due to the lack of many experimental results for some properties that are required to evaluate the change in chemical potential. Here we estimate the change in chemical potential by using an almost exact route. For methane and the hydrate, we estimate the chemical potential exactly. For water, it was necessary to assume ideal behavior of the aqueous solution of methane, which seems reasonable given the low solubility of methane. We obtained the value of the change in chemical potential for the formation of the hydrate using two different thermodynamic routes obtaining values that are fully consistent. At a temperature of 260 K, this change was of 2.4 in *k*_B_*T* units per molecule of hydrate (i.e., CH_4_(H_2_O)_5.75_). This is higher than the magnitude we found for ice formation for a supercooling of 35 K (when computed per molecule of water). We have also evaluated how this chemical potential is modified when the solubility of methane increases with respect to the value of the planar interface. By inserting methane bubbles into the solution, we have shown that the chemical potential of methane increases by about 1.6 *k*_B_*T* units, inducing an increase in the driving force for nucleation that is comparable to that produced by 20 K of additional supercooling. That explains why nucleation of the hydrate in brute force simulations is much easier when having bubbles. However, one can go beyond the bubbles. In fact, one can start from a homogeneous solution of methane having a supersaturation in the range 5–20 (reaching molar fractions up to 0.117) and then hydrate formation is observed for temperatures up to 285 K (i.e., just 10 K below the value of *T*_3_). Notice that for the TIP4P/Ice model we were never able to obtain ice in brute force studies regardless of the temperature considered. Again, the facility to nucleate hydrate now arises from the fact that, although one can not modify much neither the chemical potential of the hydrate nor that of water at a certain *T* and *p*, one can increase dramatically the chemical potential of methane by inducing supersaturation (i.e., higher concentrations than those of the planar interface) either by using bubbles or by starting from a homogeneous supersaturated solution. This increases the value of the driving force for nucleation in simulations (i.e., the value of *Δμ*_nucleation_^*^), thus reducing the free energy barrier which is proportional to the third power of the interfacial free energy and inversely proportional to the second power of *Δμ*_nucleation_^*^. Increasing *Δμ*_nucleation_^*^ by a factor of 2 reduces the free energy barrier by a factor of 4. Since the free energy barrier enters in an exponential term when determining nucleation rates by CNT, the change in *Δμ*_nucleation_^*^ provokes a dramatic change in the nucleation rates.

It was not possible to nucleate the hydrate at temperatures above 285 K in the homogeneous highly supersaturated system. This is so because the system is in a doubly metastable state (both with respect to the formation of hydrate and with respect to the formation of the bubble). The transition with the smallest value of the free energy barrier will take place first. It seems that for temperatures below 285 K the smaller free energy barrier corresponds to that of hydrate formation, whereas for temperatures above that it corresponds to bubble formation. In experiment, it is possible to nucleate hydrate at temperatures just below *T*_3_; however it is likely that in this case the process occurs via heterogeneous nucleation.

It is clear that a lot of physics can be learned by studying the solubility of methane in water. Although the system is simple, it contains a number of interesting features. In the future, it would be of interest to determine in a quantitative way the nucleation rates for this system^96,97^ in conditions where brute force simulations are useless (due to the high induction time). What is clear now is that nucleation of hydrates can be either an event with almost zero probability or an event observed in just dozens of nanoseconds. The concentration of methane is the start of this movie. Somewhat surprisingly, one can nucleate methane hydrates using the TIP4P/Ice model in brute force simulations, whereas this was never observed for this model (to the best of our knowledge) for the formation of ice.

## Supporting Information Available

Description of the order parameter used in this work for identifying hydrate (section S1) and the pressure inside methane bubbles (section S2) (PDF)
