# Supplementary material for: Solubility of Methane in Water: Some Useful Results for Hydrate Nucleation
Source: J Phys Chem B. 2022 Oct 12;126(42):8553–70. doi: 10.1021/acs.jpcb.2c04867 (PMC9623592; doi:10.1021/acs.jpcb.2c04867)
Supplement: Supplementary file 1 — jp2c04867_si_001.pdf [file jp2c04867_si_001.pdf]

Supporting Information

On the solubility of methane in water: some useful results for hydrate nucleation

Joanna Grabowska,[1,2] Samuel Blazquez,[1] Eduardo Sanz,[1] Iván. M. Zerón,[3] Jesús Algaba,[3] José Manuel Míguez,[3] Felipe J. Blas,[3] and Carlos Vega[1,*]

[1] *Dpto. Química Física I, Fac. Ciencias Químicas, Universidad Complutense de Madrid, 28040 Madrid, Spain*
[2] *Department of Physical Chemistry, Faculty of Chemistry and BioTechMed Center, Gdansk University of Technology, ul. Narutowicza 11/12, 80-233 Gdansk, Poland*
[3] *Laboratorio de Simulación Molecular y Química Computacional, CIQSO-Centro de Investigación en Química Sostenible and Departamento de Ciencias Integradas, Universidad de Huelva, 21006 Huelva Spain*

[*] Electronic mail: cvega@quim.ucm.es

S1. ORDER PARAMETER FOR HYDRATES

We shall use the order parameter of Lechner and Dellago (for the molecules of water) that we used in our previous work with ice.[1] In particular we shall consider $\overline{q_3}$ and $\overline{q_5}$. We simulated both the pure hydrate system and water with a certain amount of methane (i.e that corresponding to the solubility of the planar interface) at 400 bar and 250 K. To evaluate the $\overline{q_3}$ and $\overline{q_5}$ a 5.5 Å cutoff distance around each molecule of water (using the oxygen atom as a reference) was considered. When evaluating the size of the largest cluster two molecules at a distance less than 3.5 Å were considered as belonging to the same cluster. The values of $\overline{q_3}$ and $\overline{q_5}$ for the individual molecules of water (methane was not considered), are presented in Fig. S1. As can be seen $\overline{q_3}$ is already quite efficient[2] in separating the cloud of molecules of water in the gas phase and in the hydrate.

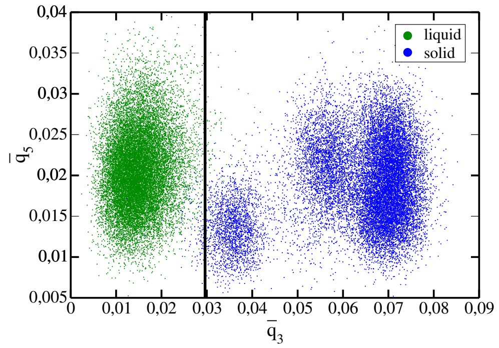

FIG. S1. Order parameters of individual molecules of water (using the oxygen atom) at 250 K and 400 bar. For each molecule of water the value of the order parameters $\overline{q_3}$ and $\overline{q_5}$ proposed by Lechner and Dellago[1] are presented. Results were obtained for the bulk hydrate (blue points) and for a bulk aqueous solution (green points). The bulk aqueous solution contain also methane molecules to reproduce the equilibrium solubility of a planar interface with the gas phase at this temperature and pressure.

Blue points correspond to the solid phase and green to the liquid. By choosing $\overline{q_3}$=0.0296 as the threshold parameter we found that 0.6 percent of the molecules of the fluid were labeled as solid, and 0.6 percent of the molecules of the solid were labeled as liquid (i.e mislabeling criteria). Thus $\overline{q_3}$ along with this threshold value is quite effective in identifying molecules of water in the solid cluster. Notice the presence of three clouds of points in the sI structure. This is because oxygen atoms occupy three different crystallographic position in the sI structure. The cloud of points of the hydrate closer to the liquid (more difficulty to identify) are those occupying the crystallographic positions of type c in the solid (with multiplicity 6).

S2. PRESSURE INSIDE METHANE BUBBLES

It is of interest to check if the bubbles of methane have the properties of a "bulk phase" of methane. For this purpose we shall present first the density profile of a certain bubble of methane at 260 K. As was discussed in Fig. 6 the density reaches a plateau. One could naively think that reaching a plateau in density indicates that the bubble has the same properties as a bulk phase having this density. We will see that this is not the case. We have computed the EOS of bulk methane at 260 K as a function of pressure to determine which will be the pressure required to achieve this density (we denoted this magnitude as mechanical pressure) - see Fig. S2. We have done that for all the bubbles considered in this work. Results are presented in Table S1.

As can be seen this pressure is different from the "thermodynamic pressure". The thermodynamic pressure is the pressure of a bulk phase of methane having the same chemical potential as methane in solution. Notice that in the Gibbsian formalism of heterogeneous curved interfaces the pressure of the internal phase should be taken as that of a bulk phase of methane having the same chemical potential as the external phase. The correct pressure for the Gibbsian description is this thermodynamic pressure. We have not computed the pressure tensor inside the bubble. However, we have estimated the mechanical pressure as that of a bulk phase of methane having the same density. For HS this

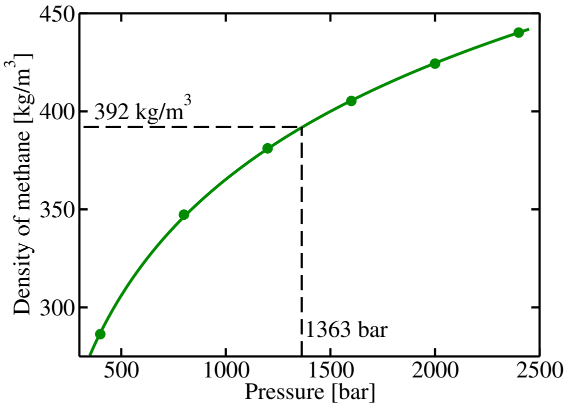

FIG. S2. Equation of state of bulk methane as a function of pressure along the isoterm of 260 K. The mechanical pressure is estimated from the known density of methane in the bubble rather than from the pressure tensor.

approach worked quite well (i.e the pressure inside the spherical phase obtained from the mechanical pressure tensor was quite similar to that obtained by assuming that its pressure can be obtained from the average density inside the spherical phase). Therefore it seems that also for the bubbles of this work mechanical and thermodynamic pressures are not identical. This may be due to the fact that the bubbles are too small to be regarded as bulk phase (in fact since the interaction of the LJ part of the potential is of 3 molecular diameters there would be strong interactions between the molecules of methane and that of water so that the bubbles are never truly bulk) or to the fact that the spherical symmetry of the bubble breaks the symmetry of a truly bulk phase.

TABLE S1. Thermodynamic and mechanical pressures (in bar) for the bubbles of methane found in this work. Radius of the bubbles R_{bubble} in nm. Thermodynamic pressure is the pressure of a bulk phase of methane having the same chemical potential as that found in the bubble (i.e. the chemical potential of methane in solution). Mechanical pressure is the pressure of a bulk phase of methane having the same density as that found in the bubble. Densities (in kg/m^3) of the bubbles are given in the last column. Differences in thermodynamic and mechanical pressures indicate that the bubble can not be regarded as a bulk phase of methane.

T/K	R_{bubble}/nm	$p^{II,\mu}$/bar	$p^{II,mechanical}$/bar	ρ_{CH_4}/ kg/m^3
250	1.77	1062	1163	385
260	1.35	1147	1408	395
	1.49	1103	1363	392
	1.98	936	1134	376
290	1.78	859	1241	365
	2.12	785	1179	360

The take home message is that one can not assume that bubbles as those shown here have the properties of bulk methane (even if the density reach a plateau). One should compute, following Gibbs the chemical potential of methane in the external phase, and to introduce in the formalism the properties of a bulk phase of methane having the same chemical potential as the external phase (and not the actual properties of the bubble).

REFERENCES

[1] Lechner, W.; Dellago, C. Accurate determination of crystal structures based on averaged local bond order parameters. *J. Chem. Phys.* **2008**, *129*, 114707.

[2] Algaba, J. M.; Acuña, E.; Míguez, J. M.; Mendiboure, B.; Zerón, I. M.; Blas, F. J. Simulation of the carbon dioxide hydrate-water interfacial energy. *Journal of Colloid and Interface Science* **2022**, *623*, 354.
